# Leucine alleviates dexamethasone-induced suppression of muscle protein synthesis via synergy involvement of mTOR and AMPK pathways

**DOI:** 10.1042/BSR20160096

**Published:** 2016-06-17

**Authors:** Xiao J. Wang, Xin Yang, Ru X. Wang, Hong C. Jiao, Jing P. Zhao, Zhi G. Song, Hai Lin

**Affiliations:** * Department of Animal Science, Shandong Agricultural University, Taian, Shandong 271018, P.R. China

**Keywords:** AMPK, glucocorticoid, leucine, mTOR, muscle cell, protein synthesis

## Abstract

Glucocorticoids (GCs) are negative muscle protein regulators that contribute to the whole-body catabolic state during stress. Mammalian target of rapamycin (mTOR)-signalling pathway, which acts as a central regulator of protein metabolism, can be activated by branched-chain amino acids (BCAA). In the present study, the effect of leucine on the suppression of protein synthesis induced by GCs and the pathway involved were investigated. *In vitro* experiments were conducted using cultured C2C12 myoblasts to study the effect of GCs on protein synthesis, and the involvement of mTOR pathway was investigated as well. After exposure to dexamethasone (DEX, 100 μmol/l) for 24 h, protein synthesis in muscle cells was significantly suppressed (*P*<0.05), the phosphorylations of mTOR, ribosomal protein S6 protein kinase 1 (p70s6k1) and eukaryotic initiation factor 4E binding protein 1 (4EBP1) were significantly reduced (*P*<0.05). Leucine supplementation (5 mmol/l, 10 mmol/l and 15 mmol/l) for 1 h alleviated the suppression of protein synthesis induced by DEX (*P*<0.05) and was accompanied with the increased phosphorylation of mTOR and decreased phosphorylation of AMPK (*P*<0.05). Branched-chain amino transferase 2 (BCAT2) mRNA level was not influenced by DEX (*P*>0.05) but was increased by leucine supplementation at a dose of 5 mmol/l (*P*<0.05).

## Abstract (precis)

Both mTOR and AMPK pathways are involved in the DEX-induced suppression of protein synthesis in muscle cells. Leucine supplementation relieves DEX-induced inhibition on protein synthesis by evoking mTOR and suppressing AMPK pathway.

## INTRODUCTION

The maintenance of skeletal muscle mass is of paramount importance for motility and systemic energy homoeostasis [1]. The control of muscle mass is determined by a dynamic balance between the anabolic and catabolic processes involving proteins [2]. Adrenal glucocorticoids (GCs) are well known to regulate an array of physiological processes, including protein metabolism, thus contributing to whole-body homoeostasis. It has been demonstrated that GCs are negative muscle protein regulators, and many pathological conditions, including muscle atrophy, are associated with an increase in circulating GCs levels [3]. In rat, muscle protein synthesis is inhibited as early as 4 h after the administration of GCs [4,5]. Shah et al. [6] showed that the injection of dexamethasone (DEX), a synthetic GC, acutely diminished protein synthesis rates to 59% of control values in skeletal muscle from young rats. The inhibition of mRNA translation initiation appears to be a major mechanism by which GCs result in the inhibition of protein synthesis [7].

Mammalian target of rapamycin (mTOR) is a crucial component of the anabolic machinery for protein synthesis, which senses and integrates signals from growth factors, environmental stress factors, nutrient availability, and energy status. mTOR has been shown to exist in two complexes (mTORC1 and mTORC2) [8]. mTORC1 is essential for the maintenance of muscle mass and function [9,10]. mTORC1 signals to ribosomal protein S6 protein kinase 1 (S6K1) and eukaryotic initiation factor 4E binding protein 1 (4EBP1), which are currently the two best-known downstream effectors of mTOR signalling, and control the protein synthetic pathway [11]. Wang et al. [12] demonstrated that the mTOR pathway is negatively regulated in the presence of excessive GCs.

AMP-activated protein kinase (AMPK), a highly sensor of cellular energy status, is activated under conditions of low intracellular ATP. AMPK acts as a major catabolic regulator in response to energy stress, in part through its inhibition of the mTORC1 pathway. The activation of AMPK directly phosphorylates both TSC2 and Raptor to inhibit mTORC1 activity by a dual-pronged mechanism [13,14]. Kimura et al. [15] also revealed that AMPK appears to provide an overriding switch linking p70s6k regulation to cellular energy metabolism. It is generally known GCs act as mediators in whole-body energy redistribution. However, whether GCs-driven protein synthesis regulation requires the cooperation of AMPK and mTOR is yet unknown.

Branched-chain amino acids (BCAA) are one of the major signals that activate mTORC1. Leucine can provide energy by conversion to ketoisocaproate, which is oxidized via the TCA cycle; leucine could decrease AMPKα phosphorylation and AMPK activity in rats [16] and C2C12 cells [17]. We thus hypothesized that BCAA supplementation could alleviate the negative effect of GCs on protein synthesis by evoking mTOR pathway with the synergy of AMPK pathway.

In the present study, we investigated whether GCs inhibit protein synthesis via the mTOR and AMPK signalling pathways, and the involvement of BCAA was examined. Herein, cultured C2C12 myoblasts were used as a model for muscle growth. DEX, a synthetic GC that is specific for the GCs receptor and delayed plasma clearance [18], was employed to induce a hyperglucocorticoid milieu. Our results indicate that GCs repress protein synthesis, likely through the involvement of both mTOR and AMPK pathways. In addition, the effects of GCs on protein synthesis and mTOR and AMPK pathways could be attenuated by leucine supplementation. Our study originally demonstrates the synergy involvement of mTOR and AMPK in the interaction between GCs and BCAA on muscular protein synthesis. This finding provides a novel insight into the metabolic perturbations associated with long-term GCs use and dietetical therapy in clinical setting.

## MATERIALS AND METHODS

### Myoblasts culture and *in vitro* treatments

C2C12 myoblasts (CCTCC) were cultured in DMEM (HyClone) supplemented with 10% fetal bovine serum (HyClone), 100 U/ml penicillin and 100 μg/ml streptomycin (Solarbio) at 37°C in a humidified atmosphere containing 5% CO_2_. When cells were 70% confluent, the proliferation medium was replaced with a differentiation medium, DMEM containing 2% horse serum. After 84 h, cells were incubated for 12 h with serum-free DMEM.

**Figure 1:**
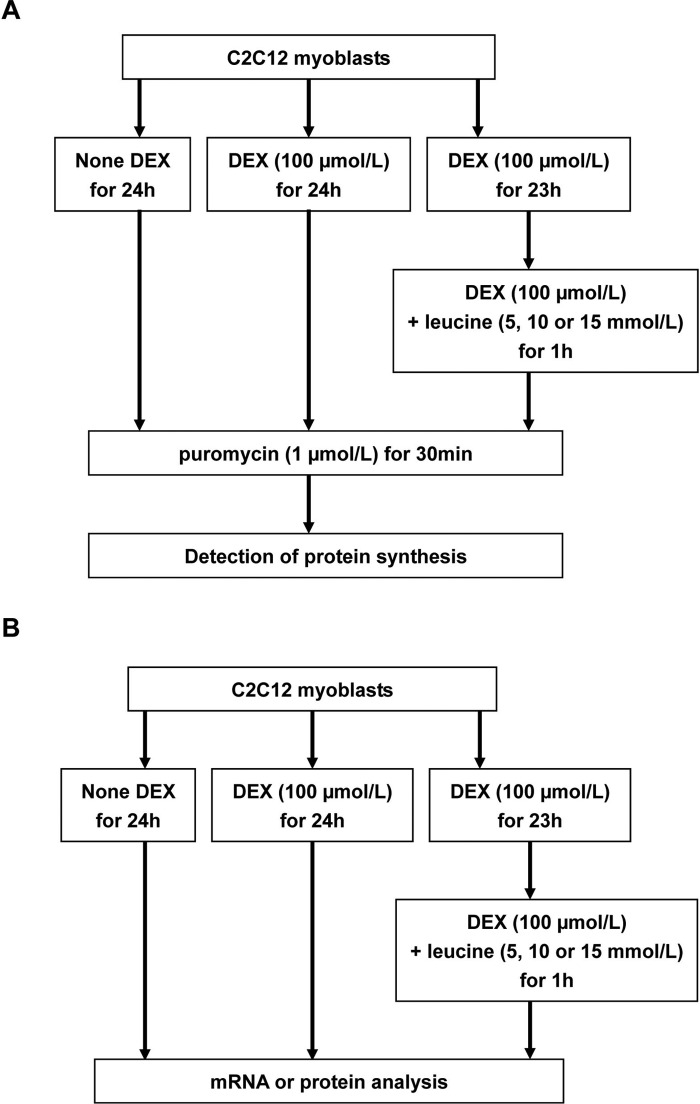
The flowchart of study design The myoblasts were incubated with or without DEX (100 μmol/l) for 24 h. At 23 h of the DEX exposure, leucine (5, 10 or 15 mmol/l) was added to DEX treated cells for the following 1 h. After this, all cells were immediately subjected to an additional 30-min puromycin exposure (1 μmol/l) and then the detection of protein synthesis (**A**), or were directly collected for mRNA and protein analysis (**B**).

After a 12-h incubation in serum-free medium, the myoblasts were exposed to DMEM-LM (Thermo) with or without DEX (100 μmol/l) for 24 h. At 23 h of the DEX exposure, leucine (Sigma) was added to DEX treated cells for the following 1 h, with a concentration of 5, 10 or 15 mmol/l. After this, all cells were immediately subjected to an additional 30-min puromycin exposure (1 μmol/l, Sigma) and then the detection of protein synthesis using an anti-puromycin antibody (Figure 1A), or were directly collected for mRNA and protein analysis (Figure 1B).

### Protein synthesis rate analysis

To measure the muscle protein synthesis rate, we used a technique involving the labelling of newly synthesized polypeptides with low concentrations of puromycin, then the detection of these proteins using an anti-puromycin antibody [19]. After DEX and leucine administration, 1 μmol/l puromycin was added to all wells, and the cells were incubated for an additional 30 min. Cells were then collected and subjected to Western blotting analysis using an anti-puromycin antibody as described below. The accumulation of puromycin-conjugated peptides into nascent peptide chains reflects the rate of protein synthesis in many different *in vitro* and *in vivo* conditions [19–21].

### Protein preparation and western blot

Protein concentration was determined using the BCA assay kit (Beyotime). The samples were boiled at 100°C for 5 min in 5× sample buffer. The protein extracts were electrophoresed in 7.5–10% SDS polyacrylamide gels (Bio-Rad Laboratories) according to the Laemmli method [22]. The separated proteins were then transferred on to a nitrocellulose membrane in Tris–glycine buffer containing 20% methanol. The membranes were blocked and immunoblotted with a 1:1000 dilution of a primary antibody including anti-puromycin (keraFAST), anti-P-mTOR (Ser^2448^), anti-mTOR, anti-P-p70S6K (Thr^389^), anti-p70S6K, anti-P-4EBP1 (Thr^37/46^), anti-4EBP1, anti-P-AMPK (Thr^172^) and anti-AMPK (Beverly, MA, USA).

The proteins were detected using either goat anti-rabbit IgG (H+L)-HRP conjugated secondary antibody (1:2000, Bio-Rad Laboratories) or HRP-labelled goat anti-mouse IgG (H+L) secondary antibody (1:1000, Beyotime) with enhanced chemiluminescence (ECL) plus western blot detection reagents (Beyotime). β-Actin was used as an internal control (Beyotime). Western blots were developed and quantified using BioSpectrum 810 with VisionWorksLS 7.1 software (UVP LLC). The protein level was quantified by normalizing total proteins with β-actin, and by normalizing phosphorylated proteins with their total pairs.

### RNA preparation and analysis

Gene expression was measured using real-time RT-PCR. Briefly, total RNA from cells was extracted using TRIzol (Invitrogen). The quantity and quality of the isolated RNA were determined using a biophotometer (Eppendorf) and agarose gel electrophoresis. Next, reverse transcription was performed using an RT reaction (10 μl) that consisted of 500 ng total RNA, 5 mmol/l MgCl_2_, 1 μl RT buffer, 1 mmol/l dNTP, 2.5 U AMV, 0.7 nmol/l oligo d(T) and 10 units ribonuclease inhibitor (TaKaRa). The cDNA was amplified in a 20 μl PCR reaction containing 0.2 μmol/l of each specific primer (Sangon) and SYBR green master mix (TaKaRa). Real-time PCR was performed at 95°C for 10 s of predenaturation, followed by 40 cycles, and each cycle consisted of denaturation at 95°C for 5 s and annealing and extension at 60°C for 40 s. Primers against β-actin was used as internal controls to normalize the differences between individual samples. The primer sequences for mouse are listed in Table 1. Standard curves were generated using pooled cDNA from the samples that were assayed, and the comparative CT method (2^−ΔΔCT^) was used to quantify mRNA expression, as described by Livak and Schmittgen [23]. All of the samples were run in duplicate, and the primers were designed to span an intron to avoid genomic DNA contamination. All samples were included in the same assay for one gene to avoid inter-assay variability.

**Table 1:** Gene-specific primers of related genes

Gene name	GenBank number	Primers position	Primers sequences (5’→3’)	Product length (bp)
β-Actin	NM_007393	Forward	ACCACACCTTCTACAATGAG	182
		Reverse	ACGACCAGAGGCATACAG	
BCAT2	NM_001243052	Forward	CCTGTTCCCTGGCTTCTATGT	100
		Reverse	GCTTCTTCTGTGGTTCTTTGGT	
MAP4K3	NM_001290345	Forward	ATTCTGTGGAGGTGGCTCTTTA	176
		Reverse	CGTGACCATTATCCGTTAGGAG	
Rheb	BC012273	Forward	TCTGTGGGAAAGTCCTCATTG	115
		Reverse	ACTCTTGACCATTTACCGTGAT	
GR	X66367	Forward	CCCATGGAGGTAGCGATTGT	100
		Reverse	TGTAAAGGCTGCCCAATGTGT	

### Statistical methods

All the data were subjected to one-way ANOVA analysis with the Statistical Analysis Systems statistical software package (Version 8e, SAS Institute). The homogeneity of variances among groups was confirmed using Bartlett's test (SAS Institute). When the primary effect of treatment was significant, differences between means were assessed by Duncan's multiple range analysis. Means were considered significantly different at *P*<0.05.

## RESULTS

**Figure 2:**
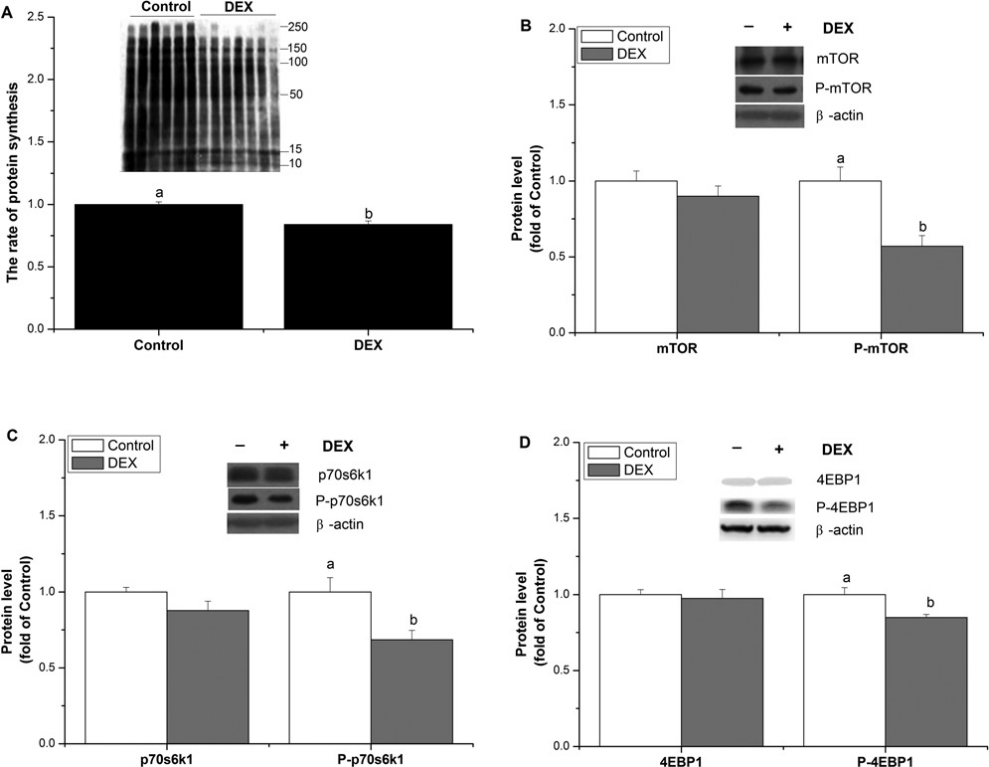
mTOR inhibition is involved in DEX-suppressed protein synthesis The effect of DEX (100 μmol/l for 24 h) on protein synthesis (**A**), and the protein expression of mTOR, P-mTOR (**B**), p70s6k, P-p70s6k (**C**), 4EBP1, P-4EBP1 (**D**) in C2C12 myoblasts. The protein level was quantified by normalizing total proteins with β-actin, and by normalizing phosphorylated proteins with their total pairs. The values shown are the means±S.E.M. (*n*=6); a, b: means with different letters are significantly different (*P*<0.05).

In cultured muscle cells, we demonstrated that the DEX treatment significantly suppressed protein synthesis (*P*<0.05, Figure 2A), as well as the phosphorylation of mTOR, p70s6k1 and 4EBP1 (*P*<0.05, Figures 2B–2D), whereas the total protein expression of mTOR, p70s6k1 and 4EBP1 was not affected by DEX (*P*>0.05, Figures 2B–2D).

**Figure 3:**
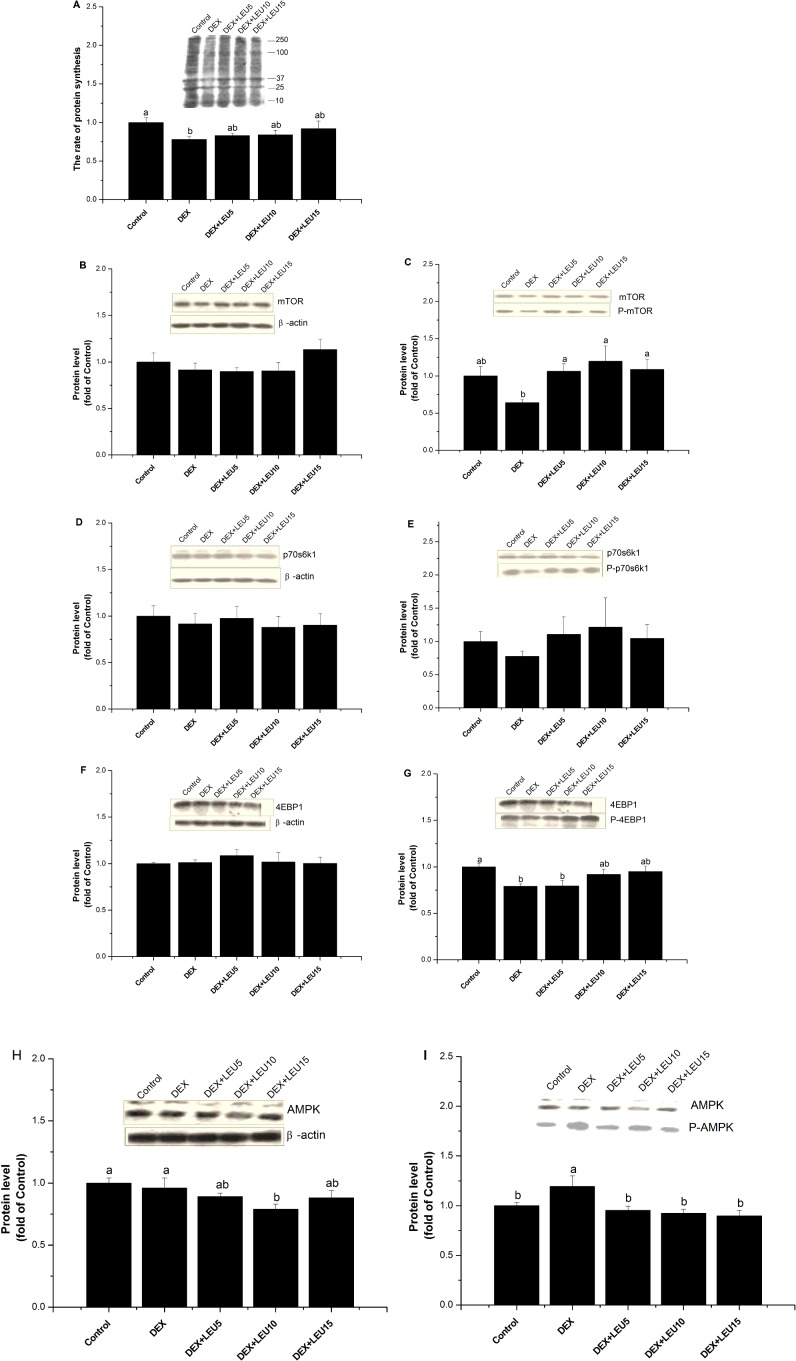
Leucine ameliorated DEX-suppressed protein synthesis by stimulating mTOR and suppressing AMPK pathway The effect of DEX (100 μmol/l for 24 h) and leucine treatment (5 mmol/l or 10 mmol/l or 15 mmol/l for 1 h) on protein synthesis (**A**), and the protein expression of mTOR (**B**), P-mTOR (**C**), p70s6k (**D**), P-p70s6k (**E**), 4EBP1 (**F**), P-4EBP1 (**G**), AMPK (**H**) and P-AMPK (**I**) in C2C12 myoblasts. The protein level was quantified by normalizing total proteins with β-actin, and by normalizing phosphorylated proteins with their total pairs. The values shown are the means±S.E.M. (*n*=6); a, b: means with different letters are significantly different (*P*<0.05).

In the presence of DEX and leucine, DEX-suppressed myoblast protein synthesis was restored to normal (Figure 3A). Leucine supplementation completely/partially alleviated the negative effect of DEX alone on both mTOR and 4EBP1 phosphorylation (Figures 3C and 3G). DEX and leucine had no effect on the protein expressions of mTOR, p70s6k1, phosphor-p70s6k1 and 4EBP1 (*P*>0.05, Figures 3B, 3D, 3E and 3F). DEX alone showed no obvious effect on the total protein level of AMPK (*P*>0.05), but DEX+leucine (10 mmol/l) treatment reduced AMPK protein expression compared with the control (*P*<0.05, Figure 3H). DEX exposure significantly enhanced the phosphorylation of AMPK compared with the control group (*P*<0.05), and this impact exerted by DEX alone was restored to the control level after supplementation with leucine (Figure 3I). These results suggest that the inhibiting effect of leucine on AMPK phosphorylation, at least partially, is due to the reduced total AMPK protein.

**Figure 4:**
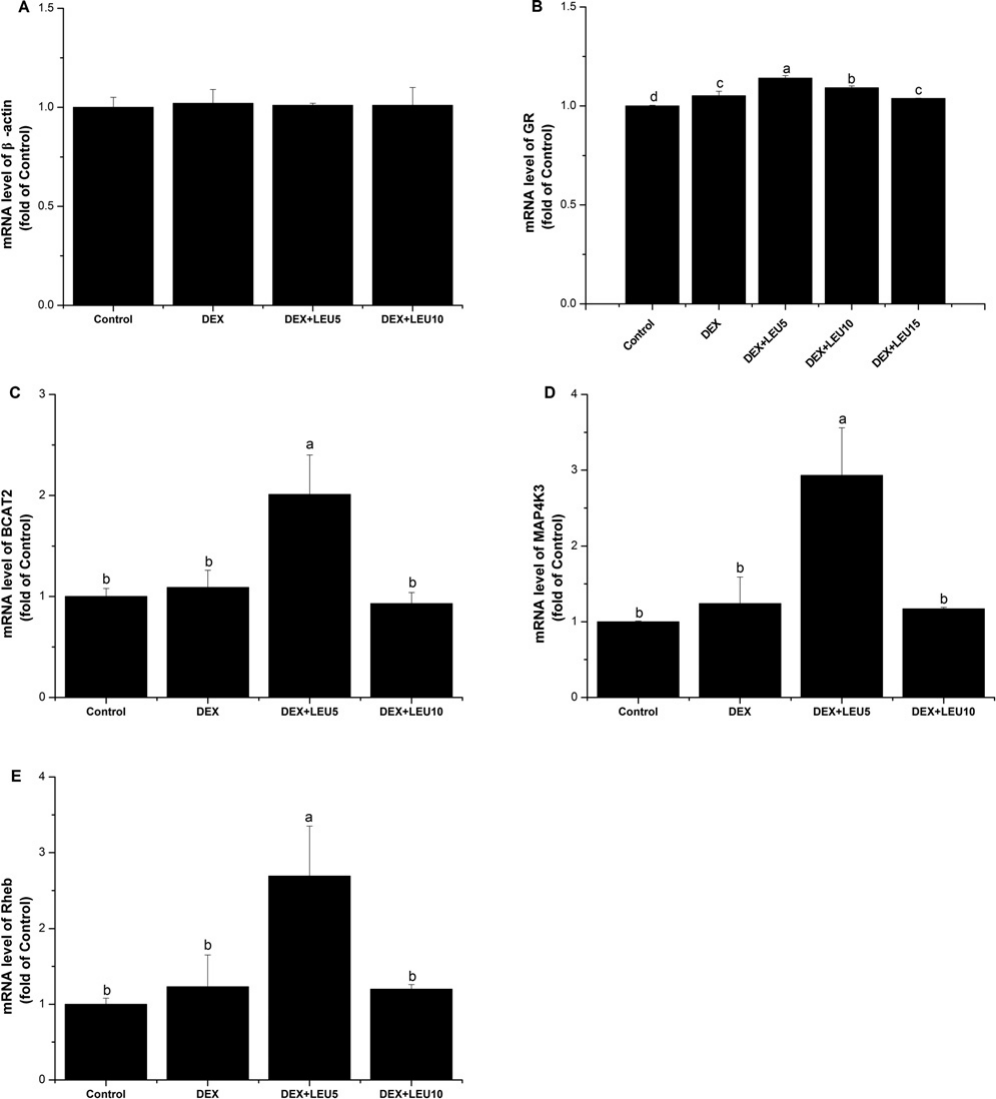
Expressions of regulators sensitive to GCs and leucine The effect of DEX (100 μmol/l for 24 h) and leucine treatment (5 mmol/l or 10 mmol/l or 15 mmol/l for 1 h) on the mRNA expression of β-actin (**A**), GR (**B**), BCAT2 (**C**), MAP4K3 (**D**) and Rheb (**E**) in C2C12 myoblasts. The values shown are the means ± S.E.M. (*n*=6); a, b, c, d: means with different letters are significantly different (*P*<0.05).

Compared with the control, glucocorticoid receptor (GR) mRNA level was increased by DEX alone (*P*<0.05), as well as DEX+leucine treatment (*P*<0.05, Figure 4B). DEX treatment had no significant influence on the mRNA expressions of β-actin, branched-chain amino transferase 2 (BCAT2), mitogen-activated protein kinase kinase kinase kinase 3 (MAP4K3) and RAS homologue enriched in brain (Rheb) compared with the control (*P*> 0.05, Figures 4A, 4C, 4D and 4E). Compared with the DEX alone, BCAT2, MAP4K3 and Rheb mRNA levels were increased in the DEX+leucine (5 mmol/l) group (*P*<0.05) but restored in the DEX+leucine (10 mmol/l) group (Figures 4C–4E).

## DISCUSSION

**Figure 5:**
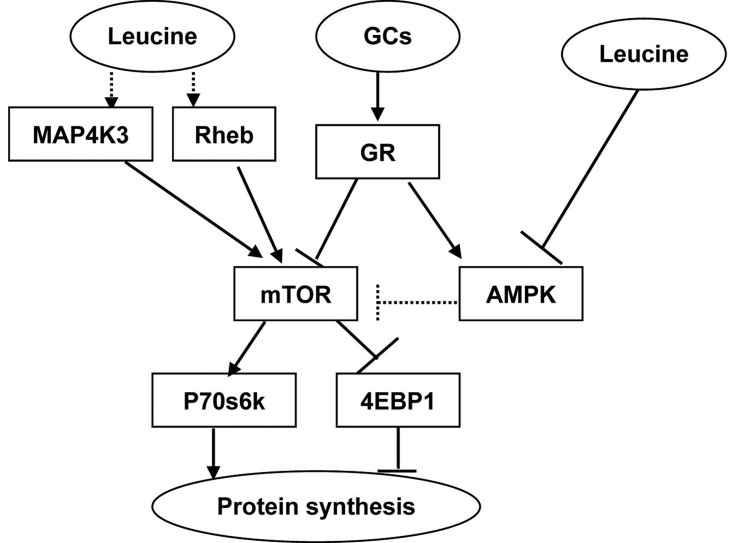
Proposed model of GCs and leucine action on protein synthesis in C2C12 myoblasts (→ stimulatory; ⊥ inhibitory; …… findings reported previously [13,14,47–49,55]) Activation of GCs suppresses myocellular protein synthesis via inhibiting mTOR and stimulating AMPK; leucine relieves GCs-induced inhibition on protein synthesis by evoking mTOR and suppressing AMPK pathway.

In the present study, we assessed the direct effect of leucine on muscle protein synthesis in the presence of GCs. The results show that the leucine supplementation could relieve the suppression effect of GCs on protein synthesis by evoking mTOR/p70s6k pathway. We firstly demonstrate that AMPK is also involved in the regulation of GCs and leucine on muscle protein synthesis. Proposed model of GCs and leucine action on protein synthesis in C2C12 myoblasts is shown in Figure 5.

### DEX retards myoblast protein synthesis

Muscle growth is largely due to the balance of muscle protein synthesis and degradation. In the present study, we determined the protein synthesis rate of cultured C2C12 myoblasts by using puromycin to label the newly-synthesized polypeptides [21]. We demonstrated that DEX-suppressed protein synthesis, in line with previous findings *in vivo* [4–6]. This may explain the involvement of GCs in catabolism/anabolism disorders, which are associated with a number of pathological conditions, including muscle atrophy [24–26].

### mTOR inhibition is involved in DEX-suppressed protein synthesis

mTOR acts as a critical mediator that controls protein synthesis at the transcriptional and translational levels, by sensing and integrating signals from nutrients and energy. mTOR activation up-regulates the translational machinery and promotes protein translation [27]. Previous studies have demonstrated the role for GCs in the modulation of mTOR signalling and in the regulation of protein synthesis. Rannels et al. [7] revealed that the inhibition of protein synthesis by GCs is most probably associated with the inhibition of mRNA translation initiation. GCs suppressed mTOR pathway by dephosphorylating p70s6k and 4EBP1 in skeletal muscle cells [6,28–30], which are currently the two best-known downstream effectors of mTOR signalling, and control the protein synthetic pathway [11]. In the present study, the decreased phosphorylation of mTOR and p70s6k and 4EBP1 by DEX indicated that DEX-suppressed muscle protein synthesis by inhibiting the mTOR signalling pathway. The observation is consistent with the results obtained by Long et al. [31], who reported that DEX inhibits the stimulation of muscle protein synthesis and the phosphorylation of p70s6k.

### Leucine ameliorated DEX-suppressed protein synthesis by stimulating mTOR pathway

mTOR regulates multiple cellular functions, including translation, in response to nutrients, especially BCAA. Bolster et al. [32] and Deldicque et al. [33] reported that amino acids stimulate muscle protein synthesis partially through the activation of the mTOR pathways. Kimball and Jefferson [13] also revealed that BCAA mediates translational control of protein synthesis. Leucine and other members of the BCAA family are the dominant players in the amino acids-induced regulation of p70s6k [34–36]. Although insulin alone can increase muscle protein synthesis in animals, amino acids (particularly leucine) appear to have much more potent anabolic effect [13,37–39]. The administration of leucine after fasting or amino acids starvation stimulates protein synthesis and promotes the phosphorylation and activation of S6K1 via the rapamycin sensitive mTOR in skeletal muscle [37]. GCs could inhibit mTOR activity [40]. Therefore, we tested that whether leucine supplementation could relieve the inhibition of mTOR by GCs. The present result indicated that DEX-induced myoblast protein synthesis was restored to 83–92% of normal by leucine supplementation. Meanwhile, leucine supplementation completely/partially removed the inhibition of DEX on mTOR and 4EBP1 phosphorylation. The result suggests that leucine relieves the negative effect of DEX on protein synthesis by evoking mTOR pathway.

The effect of GCs is mediated by the ligand-dependent activation of GR. Upon binding GCs, the activated GR acts as a transcription factor, translocating into the nucleus and controlling the level of target gene expression and modulating intracellular signalling pathways [41–43]. GR is mandatory for muscle atrophy in response to GCs excess both *in vitro* [44] and *in vivo* [45]. On the contrary, muscle-specific GR-knockout mice are resistant to the atrophy induced by GCs [46]. GCs inhibit mTOR activity via GR [40]. Herein, we found that GR mRNA abundance was elevated by DEX. Moreover, leucine supplementation further up-regulated GR mRNA level, suggesting that increased abundant mRNA level of GR is a feedback effect of GC and leucine.

BCAT2, a mitochondrial enzyme catalysing the first reaction in the catabolism of BCAA [46], is a critical determinant of cellular BCAA content in skeletal muscle. GCs inhibit mTOR activity by evoking the activity of BCAT [40]. In the present study, BCAT2 mRNA level was not influenced by DEX but was increased by leucine supplementation at a dose of 5 mmol/l. The result may imply that BCAT2 is not the only way involved in the regulation of GCs and leucine on mTOR. The increase in BCAT2 mRNA level with 5 mmol/l leucine was lost with 10 mmol/l leucine, suggesting a substrate inhibition characteristic of BCAT2.

MAP4K3 is an upstream amino acids-sensitive regulator of mTORC1 signalling [47]. In primary human fibroblasts, knockdown of MAP4K3 resulted in a significant attenuation in leucine-induced mTORC1 signalling [48]. Rheb is also known to be critical elements in the pathway that links amino acids availability to mTORC1 activation [49]. In the present study, the mRNA expression of Rheb and MAP4K3 were enhanced when 5 mmol/l leucine was supplemented, compared with DEX alone. However, this transcriptional stimulation of Rheb and MAP4K3 was not observed after 10 mmol/l leucine supplementation. The irregularity of leucine action on mRNA expression indirectly confirmed that leucine may exert it action on Rheb and MAP4K3 via a post-transcriptional regulation. This speculation is consistent with studies of Yan et al. [50] and Tee et al. [51] who reported that Rheb farnesylation and MAP4K3 phosphorylation were required for mTORC1 activation. Further works on translational and post-translational modification are required.

### AMPK is involved in the activation of mTOR by leucine

AMPK modulates metabolism for cellular energy demand by responding to changes in the AMP/ATP ratio [52–54]. AMPK suppresses protein synthesis in rat skeletal muscle through the down-regulation of mTOR signalling [55]. AMPK activation can phosphorylate both TSC2 and Raptor, resulting in the depression of mTORC1 signalling [13,14]. AMPK appears to provide an overriding switch that links p70s6k regulation to cellular energy metabolism [15]. In the present study, the increased phosphorylation level of AMPK by DEX treatment indicated that the stimulated AMPK pathway by DEX. Leucine supplementation, however, down-regulated the phosphorylation of AMPK. The result suggests that AMPK synergizes mTOR underlying in the regulation of GCs and leucine on muscle protein synthesis. These novel observations of the synergy effect of AMPK and mTOR pathways are consistent with the results of Lang et al. [56] and Du et al. [17], who reported that leucine stimulates mTOR, at least partially, through the inactivation of AMPK.

In conclusion, both mTOR and AMPK pathways are involved in the DEX-induced suppression of protein synthesis in muscle cells. Leucine supplementation relieves DEX-induced inhibition on protein synthesis by evoking mTOR and suppressing AMPK pathway.

## Abbreviations

AMPK: AMP-activated protein kinase
BCAA: branched-chain amino acids
BCAT: branched-chain amino transferase
DEX: dexamethasone
4EBP1: eukaryotic initiation factor 4E binding protein 1
GCs: glucocorticoids
GR: glucocorticoid receptor
MAP4K3: mitogen-activated protein kinase kinase kinase kinase 3
mTOR: mammalian target of rapamycin
p70s6k: ribosomal protein S6 protein kinase
Rheb: RAS homologue enriched in brain
